# Invasive Fungal Infection with *Absidia Corymbifera* in Immunocompetent Patient with Electrical Scalp Burn

**Published:** 2018-05

**Authors:** Prashant Moon, N Jithendran

**Affiliations:** 1 Krishna Hospital and Research Center, Gurunanak Pura, Nainital Road, India; 2 Aware Global Hospital, Hyderabad, India

**Keywords:** Fungus, *Absidia corymbifera*, Immunocompetent, Scalp, Electrical burn

## Abstract

Invasive fungal infection in burn injury is caused by inoculation of fungal spore from patient skin, respiratory tract or from care giver. The risk factors for acquiring fungal infection in burns include age of burns, total burn size, full thickness burns, inhalational injury, prolonged hospital stay, late surgical excision, open dressing, central venous catheters, antibiotics, steroid treatment, long-term artificial ventilation, fungal wound colonization, hyperglycemic episodes and other immunosuppressive disorders. Invasive fungal infection with *Absidia corymbifera* is rare opportunistic infection encountered in patient with burn injury. The key for treatment is early clinical diagnosis, wide and repeated debridement and systemic and local antifungal treatment. We describe a case of invasive fungal infection with *A. corymbifera* in a patient with post-electrical scalp burn with late presentation after 10 days of injury in an immunocompetent patient.

## INTRODUCTION

Burn wound infection is primarily caused by bacteria (70%) followed by fungi (20-25%), and virus (5-10%). Fungi causes burn wound infection as part of monomicrobial or polymicrobial infection. Zygomycosis is a rare aggressive soft tissue infection and opportunistic infections.^1^ The risk factors for acquiring fungal infection in burns include age of burns, total burn size, body surface area (BSA) (30-60%), full thickness burns, inhalational injury, prolonged hospital stay, late surgical excision, open dressing, artificial dermis, central venous catheters, antibiotics, steroid treatment, long-term artificial ventilation, fungal wound colonization, hyperglycemic episodes and other immunosuppressive disorders.^2^^-^^5^

*Absidia corymbifera* is a saprophytic organism with worldwide distribution that is isolated from soil as well as decaying vegetation and grass. It is uncommon pathogen representing only 2% to 3% of all Zygomycetes infection in humans. The organism is opportunistic, rarely infecting the immunocompetent although such cases have been reported.^6^ Zygomycetes characteristically invade blood vessels, leading to thrombosis and infarction, with subsequent tissue necrosis and eschar formation. The resultant necrotic tissue favors further growth of the fungus and limits penetration of systemic antifungal agents.^7^

This ability to invade intact skin through protiolytic enzyme and angioinvasive propensity is associated with high mortality. It demands a multidisciplinary approach in burn patients with presence of zygomycosis, associated with invasion of healthy skin.^8^ ^Corymbifera^ is the only species of the genus *Absidia* recognized as a human pathogen. It accounts for perhaps 2 to 3% of culture-confirmed cases of zygomycetes infection.^9^ Suspicion on clinical finding, extensive debridement, broad spectrum systemic anti fungal drugs and coverage of wound with durable cover after confirmation of elimination of disease is required for the treatment of invasive fungal infection.

## CASE REPORT

**Fig. 1:**
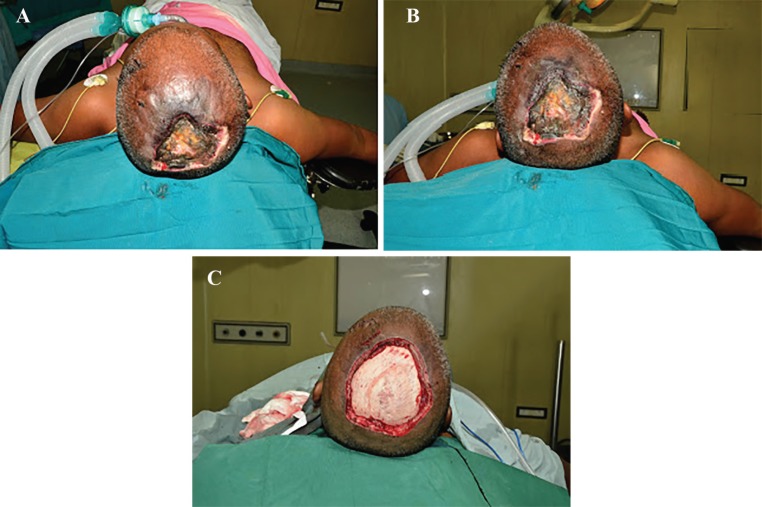
**A)** Patient on admission at first debridement. **B)** Patient on admission at first debridement. **C)** Patient after first debridement.

**Fig. 2:**
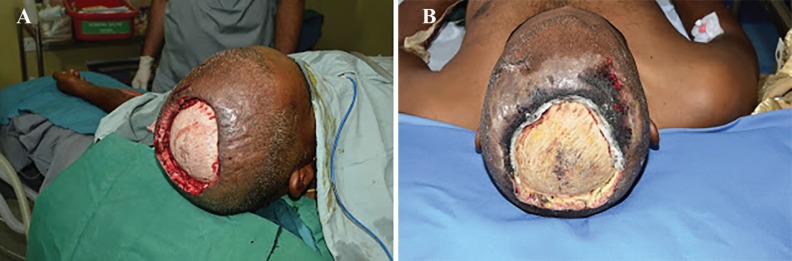
**A)** Patient after first debridement. **B)** Development of fungal infection with fungal hyphae seen in the wound

A 32 year-old male sustained high voltage electrical contact burn over scalp. Patient was treated with dressing for 10 days in other hospital. Patient was presented in our casualty with fever, raised counts and infected wound over scalp. Our patient was not having any history of immune compromise. Patient had undergone debridement of scalp with gross clear margin (Figure 1A-C). Patient had a sustained fever and raised counts even after adequate debridement (Figure 2A). On clinical examination after two days, there was evidence of hyphae over wound margin and gangrenous changes over scalp surrounding the wound (Figure 2B). 

**Fig. 3:**
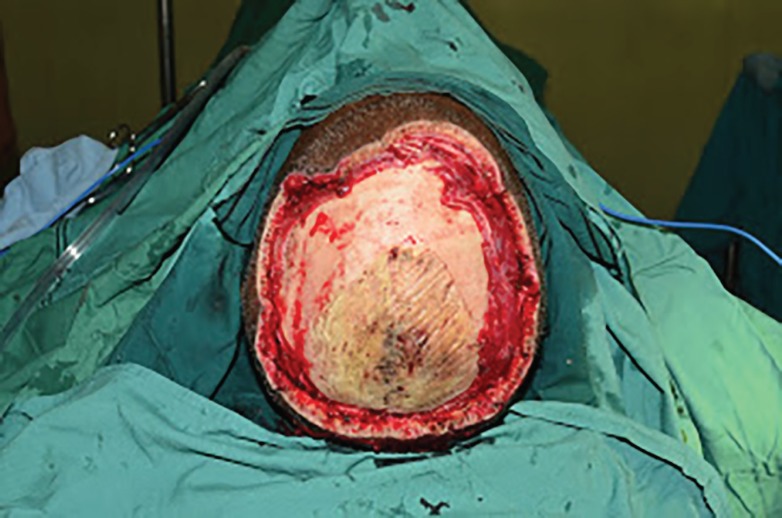
Aggressive radical debridement

**Fig. 4:**
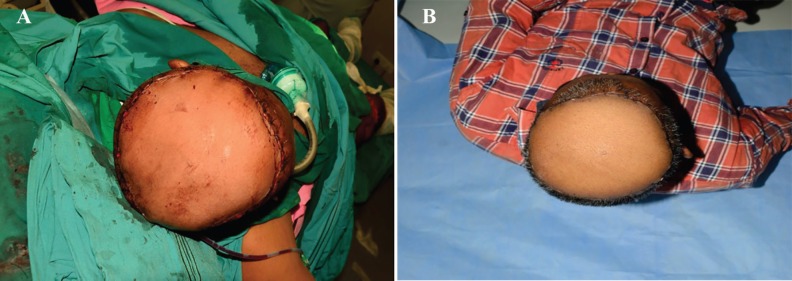
**A)** Flap coverage with anterolateral thigh flap. **B)** Well settled flap

On clinical suspicion of invasive fungal infection (zygomycosis), injection of amphotericin B was started and patient was taken for wide debridement. Tissue biopsy was taken and sent for histopathology examination. Outer bony cortex chiseling was done with high speed diamond burr. Histopathology report of specimen came out to be *A. corymbifera*. Intravenous amphotericin B injection was continued for 4-week period since diagnosis to complete healing. After two weeks of anti-fungal treatment, clinical condition of patient improved. After confirmation of absence of fungal infection, scalp defect was covered with free anterior lateral thigh flap (Figure 3). Recipient vessels were superior temporal artery and vein. In post-operative period, patient was on intravenous amphotericin B injection for 2 weeks. Post-operative period was uneventful (Figure 4A-B). 

## DISCUSSION

Cutaneous Invasive fungal infection is devastating condition in which delay in diagnosis and treatment may lead to high morbidity and mortality. Cutaneous zygomycosis remains under diagnosed despite being frequently encountered in humans; zygomycosis is caused by mucorales which contain genera mucor, rhizopus, *Absidia*, cunnighmella, rhizomucor, apophysomyces, etc. Rhino cerebral and pulmonary zygomycosis is most common while invasive skin infection is less common. Invasive skin infection with Absidia is very rare entity with only few cases reported.^10^

Morbidity and mortality with zygomycosis is very high, if diagnosis and subsequent treatment is delayed. Primary breakdown in the integrity of the cutaneous barrier such as trauma, surgical wounds, needle sticks, or burns in immune-comprised patient leads to zygomycosis. Zygomycosis occurs rarely in immune-competent hosts. This mucorales are associated with angioinvasive disease, often leading to thrombosis, infarction of involved tissues, and tissue destruction mediated by a number of fungal proteases, lipases, and mycotoxins. If the diagnosis is not made early, dissemination often occurs.^11^

Early administration of systemic antifungal drug and early debridement of infected tissue is required for management of invasive fungal infection. There should be repeated inspection of burn wound to look for residual fungi infection. Flap coverage should be given to the wound only after confirmation of elimination of infection.^7^ Although invasive fungal infection is rare in burn patient, there should be high degree of suspicion because mortality associated with invasive fungal infection is high. Early diagnosis, prompt treatment with systemic and local antibiotic and wide debridement is important. Coverage of wound should be done after confirmation of absence of fungal infection.

## CONFLICT OF INTEREST

The authors declare no conflict of interest.
